# Measuring progress on health and well-being in the Eastern Mediterranean Region via voluntary national reviews, 2016–2021: What does the data reveal?

**DOI:** 10.1371/journal.pgph.0002838

**Published:** 2024-07-18

**Authors:** Ruth M. Mabry, Henry V. Doctor, Mina N. Khair, Maha Abdelgalil, Arash Rashidian

**Affiliations:** 1 Global Health Consultant, Muscat, Oman; 2 Department of Science, Information and Dissemination, World Health Organization Regional Office for the Eastern Mediterranean, Cairo, Egypt; 3 WHO Health Emergency Programme, World Health Organization Regional Office for the Eastern Mediterranean, Cairo, Egypt; University of Washington Department of Global Health, UNITED STATES OF AMERICA

## Abstract

**Background:**

Country **s**ubmission of Voluntary National Reviews is the formal mechanism to report on progress of the Sustainable Development Goals (SDGs). Despite strong political commitment to strong information systems, large data gaps exist in the Eastern Mediterranean Region.

**Methods:**

This study aims to review reports submitted by countries in the region to assess the comprehensiveness of reporting on the health-reported SDG targets and indicators. We conducted a content analysis of reports submitted between 2016 and 2021 of 18 countries of the region. The review focused on progress on the SDGs by assessing i) the reporting on the 50 health-related targets and indicators ii) data availability using the WHO reporting framework, and iii) data availability based on source of information. Spreadsheets were developed and used to extract data and facilitate content analysis.

**Results:**

All reports confirmed that SDG monitoring and reporting mechanisms have been established, however, only 11 reported on all 17 SDGs and 8 explicitly mentioned country specific 2030 targets. Many reports identified data availability as a key challenge to SDG monitoring; for the health SDG, data availability ranged from 48% to 93% among the five countries reporting this figure. Comprehensiveness of reporting varied by type of indicator (maternal, child and infant mortality were the most common) and by country income level (greater reporting by high income countries).

**Conclusions:**

Significant work remains to enhance information systems across the region to monitor progress and guide actions to achieve the health-related SDGs. Strengthening health information systems regulatory frameworks, data collection capacities including strengthening civil registration and vital statistics and population-based surveys are key steps to enhancing access to quality data which in turn can contribute to achieving the health-related SDGs.

## Introduction

In 2015, the United Nations (UN) General Assembly adopted the 2030 Agenda on Sustainable Development [1]. Building on the Millennium Development Goals, this new agenda outlined an ambitious plan addressing 17 Sustainable Development Goals (SDGs) and 169 targets to be achieved by 2030. Countries regularly prepare Voluntary National Reviews (VNRs) to the United Nations High-level Political Forum on Sustainable Development, the formal mechanism to report on progress and share lessons learned on SDG implementation [2].

SDG 3 on health and well-being is a key indicator of success in achieving the 2030 Agenda [3]. Progress on the health-related SDGs is slow around the world [4]. Prior to the COVID-19 pandemic, progress was made on only half of the 50 indicators in the 22 countries of the Eastern Mediterranean Region (EMR; Afghanistan, Bahrain, Djibouti, Egypt, Iran, Islamic Republic of, Iraq, Jordan, Kuwait, Lebanon, Libya, Morocco, occupied Palestinian territory, Oman, Pakistan, Qatar, Saudi Arabia, Somalia, Sudan, Syrian Arab Republic, Tunisia, United Arab Emirates and Yemen); the rate of progress on seven of them is too slow to meet global targets [5,6]. By 2023, progress is nearly at a standstill with marked setbacks across most indicators on health, health risks and determinants and access to health services in the region [7].

Access to quality data can contribute to achieving the health-related SDGs; better data and information is essential for evidence-informed decision-making, proper management and rational resource allocation and for monitoring and reporting [8,9]. Quality reliable data requires a well-functioning national health information system. However, limited availability of data in countries of the region, especially lack of trends and disaggregated data–one in five indicators are not available and one in four indicators are from 2019 or earlier–is a major challenge for monitoring and reporting progress [7]. Despite, strong political commitment to having national health information systems that respond to national needs, large data gaps constrain the ability for policy-makers to take evidence-based public health actions [6,10–12].

A regional review of 60 voluntary national reviews submitted by countries in the European Region reported weak national and subnational health information systems as a core challenge [13]. Similarly, a review of reports submitted by 18 countries in the Eastern Mediterranean Region identified data availability as one of the most common challenges to SDG implementation [14]. Building on this regional review, this study aims to assess the comprehensiveness of reporting on the health-reported SDG targets and indicators and reflect on country-level priorities for monitoring them.

## Methods

Similar to our earlier review [14], here we reviewed the latest voluntary national reviews from 18 countries of the EMR submitted between 2016 and 2021 and available on the UN website (https://sustainabledevelopment.un.org/vnrs/; accessed 10 November 2022). The review focused on progress on the SDGs, especially the health-related ones. Tracking progress involved assessing the reporting on the 50 health-related SDG targets and indicators; including 32 indicators associated with the 17 SDG3 targets. World Bank classifications (low- middle- and high-income countries, respectively) were used for comparative purposes, as common practice in regional assessments (Low income: Afghanistan, Sudan, and Syria, Middle income: Egypt, Iraq, Jordan, Lebanon, Libya, Morocco, occupied Palestinian territory, Pakistan, and Tunisia, High income: Bahrain, Kuwait, Oman, Qatar, Saudi Arabia and United Arab Emirates; see: https://data.worldbank.org/) [15–17]. We also assessed data availability using the WHO reporting framework of 47 indicators across 5 domains (mortality, morbidity, means of implementation, risk factors and determinants) [6], comparing the availability of data as reported by WHO Regional Office for the Eastern Mediterranean (EMRO) for the same year as the latest country report. These data are provided and validated by countries annually. We also assessed availability of data based on source of information recognizing that well-functioning health information systems would be able to provide the necessary information to report on 29 of the 32 SDG 3 Health indicators: civil registration and vital statistics system (11), country national survey (6), the disease surveillance system (3) and the routine health information system (9) (S1 Table) [8].

Spreadsheets were developed and used to extract data and facilitate content analysis in terms prioritization of SDGs and reporting of SDG health-related indicators. The data that support the findings of this study are available on request from the corresponding author.

## Results

**Fig 1:**
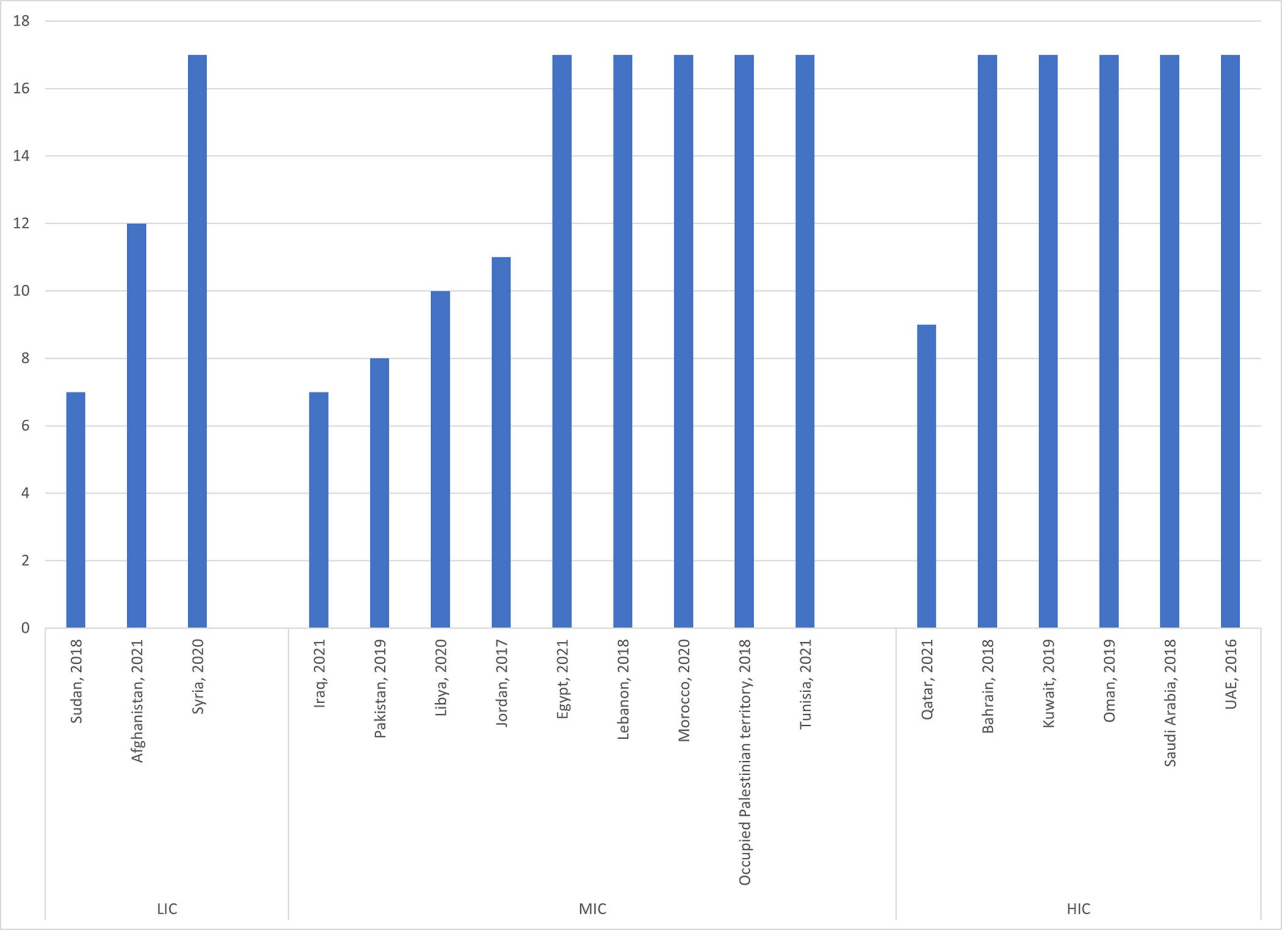
Number of SDGs prioritized by countries in their VNRs (N = 18).

All countries reported that mechanisms have been established to monitor and report on progress on the SDGs. Eleven countries reported on all 17 SDGs, the remaining reported on seven to 12 SDGs (Fig 1). The comprehensiveness of reporting across SDGs was associated with country income levels so that higher income countries tended to report on more SDGs. 17 countries reported that targets and indicators had been prioritized and adapted to the country context. However, only 8 countries explicitly mentioned country targets for 2030 for at least some of the indicators. Egypt, for example, identified SDGs “Push” targets across all the SDGs. Morocco described the level of advancement for each target and explained their improvement plan. Libya mentioned the targets for the 10 SDGs for which they reported on. Other countries had a more limited approach. For example, Jordan identified targets for SDGs 1 No poverty, 6 Water and sanitation, 7 Clean energy and 13 Climate action; Lebanon only mentioned targets for SDG 6 Waer and sanitation, 7 Clean energy, 13 Climate action and 15 Life on land. Pakistan grouped the SDGs into 3 categories; SDGs for immediate attention (2 Zero hunger, 3 Health, 4 Education, 7 Clean energy, 8 Economic growth, and 16 Peace) identified specific targets.

Nine countries (Afghanistan, Egypt, Iraq, Jordan, Morocco, occupied Palestinian territory, Oman, Pakistan, and Qatar) reported on the availability of data to report on the SDG indicators; however, description on how a data gap analysis was conducted was not included (Table 1). Of the seven countries (Egypt, Iraq, Jordan, Morocco, occupied Palestinian territory, Oman, and Qatar) that reported results of data gap analyses, Morocco and Qatar, were the only ones reported more than 50% data availability for the 244 SDG indicators. The availability of data for SDG 3 ranged from 48% to 93% among the five countries (Jordan, Morocco, Oman, occupied Palestinian territory, and Qatar) reporting this figure with Oman was the only country which reported higher availability of data for SDG 3 than for all the other Goals. Fourteen countries mentioned limited data availability as one of the challenges to monitoring and reporting. They usually described the limitations in terms of national human resource capacity to collect and/or analyse information. Additional challenges mentioned included low data quality, lack of statistical tools, limited transparency in data dissemination, and lack of data disaggregation.

**Table 1:** Data availability as described in the voluntary national reviews, EMR.

Country (year)*	Percent of SDG 3 indicators available (Percent of all SDGs indicators available)
Afghanistan, 2021	NA, however, data gap analysis was conducted
Egypt, 2021	NA (Overall: 48%)
Iraq, 2021	NA (36%)
Jordan, 2017	54% (40%, range 0–67%)
Morocco, 2020	51.9% (NA)
Occupied Palestinian territory, 2018	56% (45%, range 0–77%)
Oman, 2019	89% (41%; range: 7 to 89%)
Pakistan, 2019	NA, however, data gap analysis was conducted
Qatar, 2021	93% (NA, range: 64–100%)

*Year of most recent VNR; NA = not available.

**Fig 2:**
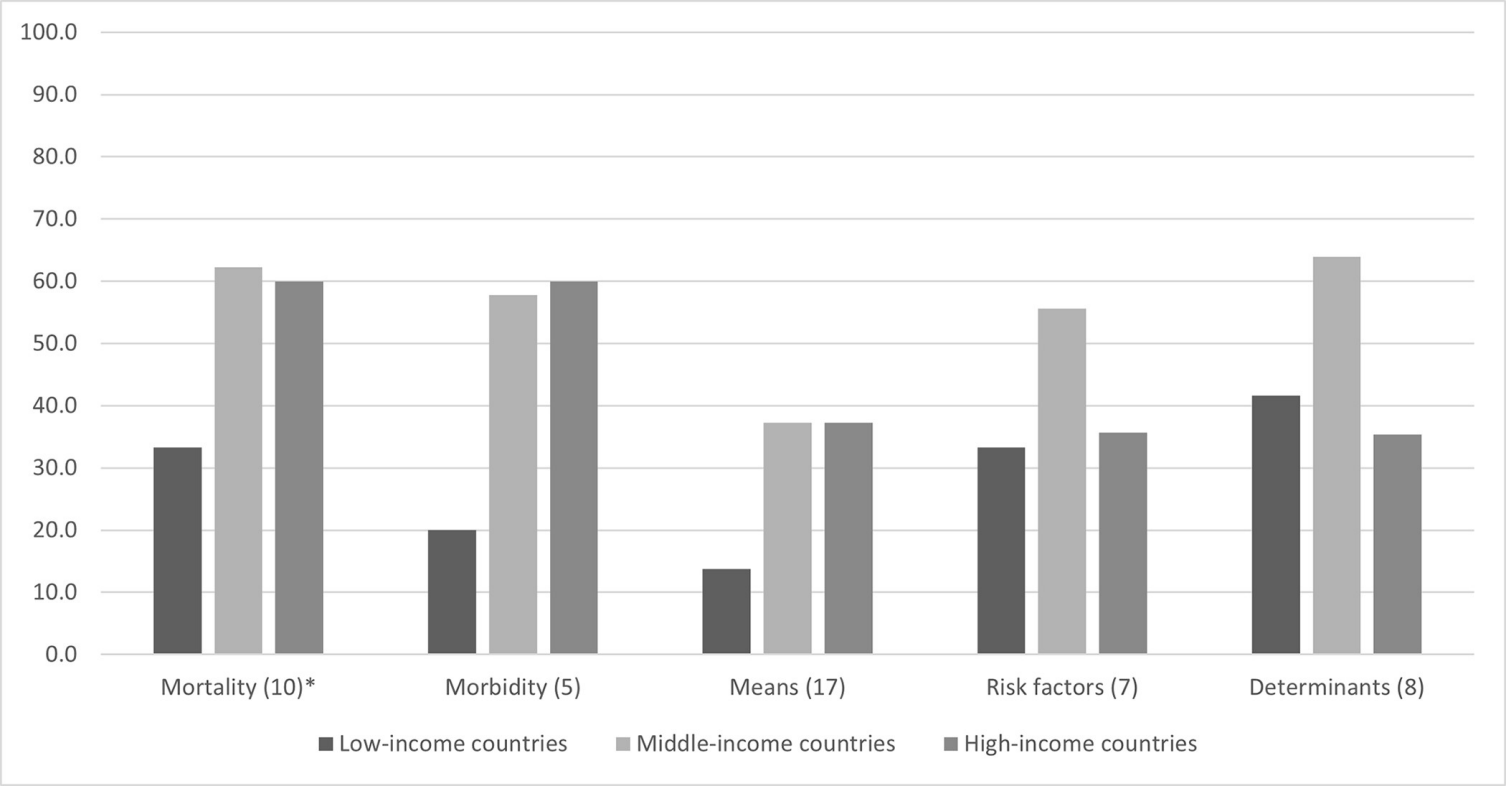
Percent of reported SDG health-related indicators in EMR Voluntary National Reports, by income levels. *Number of SDG targets for each category: Mortality (3.1, 3.2, 3.2, 3.4, 3.6, 3.9); Morbidity (3.3); Means (3.5, 3.7, 3.8, 3.a, 3.b, 3.c, 3.d); Risk factors (2.2, 6.1, 6.2, 11.6, 16.1, 16.2); Determinants (1.1, 4.1, 4.6, 5.2, 5.6, 8.5); Number in parenthesis is total number of indicators for the set of targets. Low income countries: Afghanistan, Sudan, and Syria, Middle income countries: Egypt, Iraq, Jordan, Lebanon, Libya, Morocco, Pakistan, Palestine and Tunisia, High income countries: Bahrain, Kuwait, Oman, Qatar, Saudi Arabia and United Arab Emirates.

The comprehensiveness of reporting progress on specific health-related indicators varied by type of indicator and by country income levels. The highest proportion of indicators reported were by high income countries for mortality and morbidity indicators (60%); the lowest were the means for implementation reported by low-income countries and middle-income countries (MICs); 14.7% and 20.6%, respectively. Middle- and high-income countries reported a higher percentage of targets across the five areas compared to low-income countries. Unlike with disease indicators, middle-income countries reported on more targets in the areas of risk factors (50.0% and 35.7%, respectively) and determinants compared to high-income countries (52.1% and 35.4%, respectively) (Fig 2).

**Fig 3:**
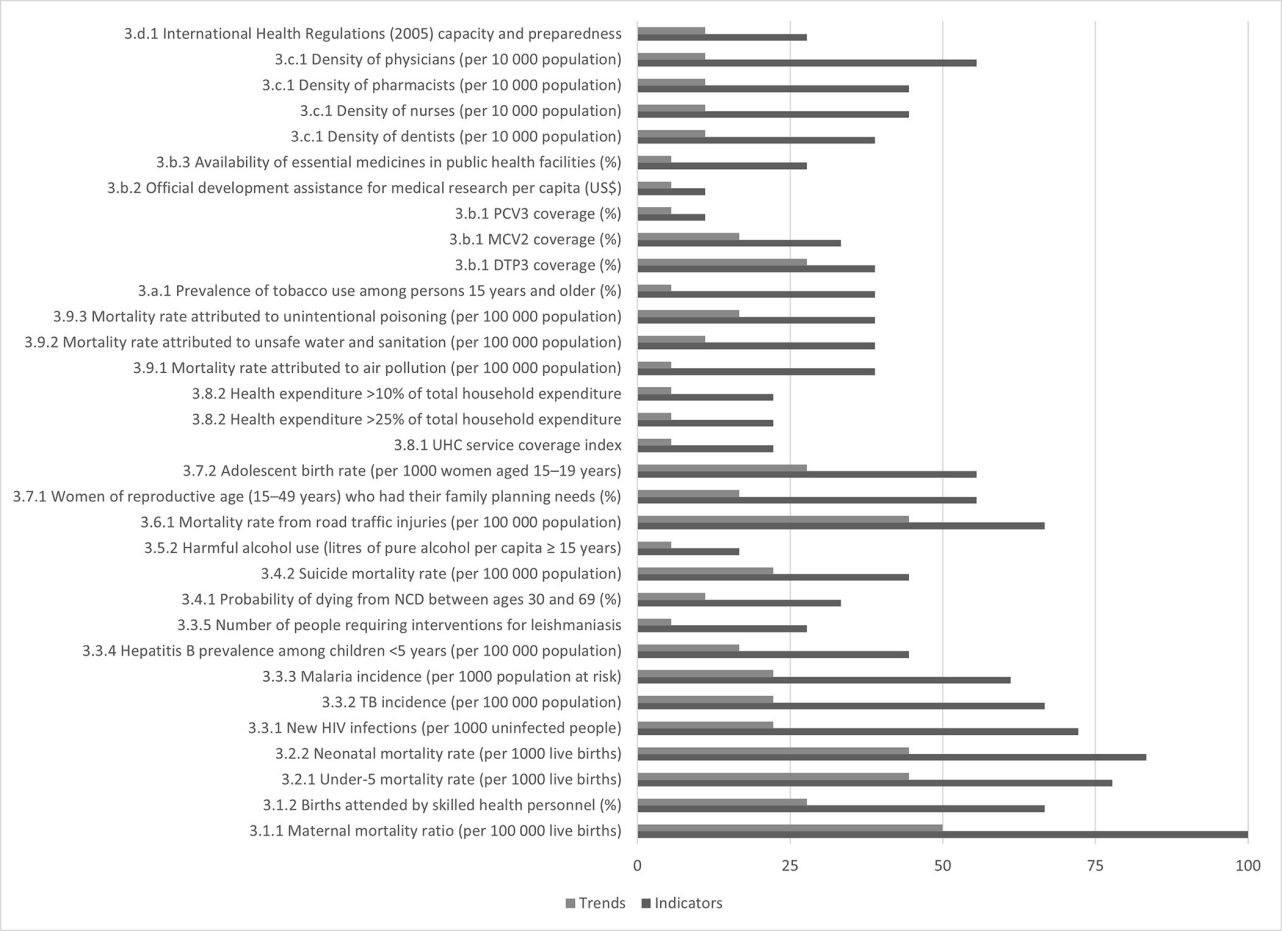
Proportion of VNRs reporting SDG3 indicators and trends.

The most commonly reported indicators were those related to SDG 3 including 3.1.1. maternal mortality ratio (100%), 3.2.2 neonatal (83%) and 3.2.1 under-5 mortality rates (78%) (Fig 3 and S1 and S2 Tables). The other indicators that reported by at least 70% of the VNRs included access to 6.1.1 improved drinking water (78%), 3.3.1 new HIV infections (72%) and 8.5.2 total unemployment rates (72%). Reporting on progress with at least two data points was less common; the most common indicator for reporting trends figures was for maternal mortality ratio (50%).

Qatar was the only country that reported on trends across all the SDG 3 indicators; all other countries provided trend data for 9 or less SDG 3 indicators (S1 Table). On average, countries provided data for about 11 indicators linked to SDG 3 targets (S1 Table) and 6.4 indicators to non-SDG 3 indicators (range 0–12, S2 Table). Data available from the WHO/EMRO reports (S1 and S2 Tables) often were not included in the reports even though these figures are routinely reported to WHO/EMRO by countries.

**Fig 4:**
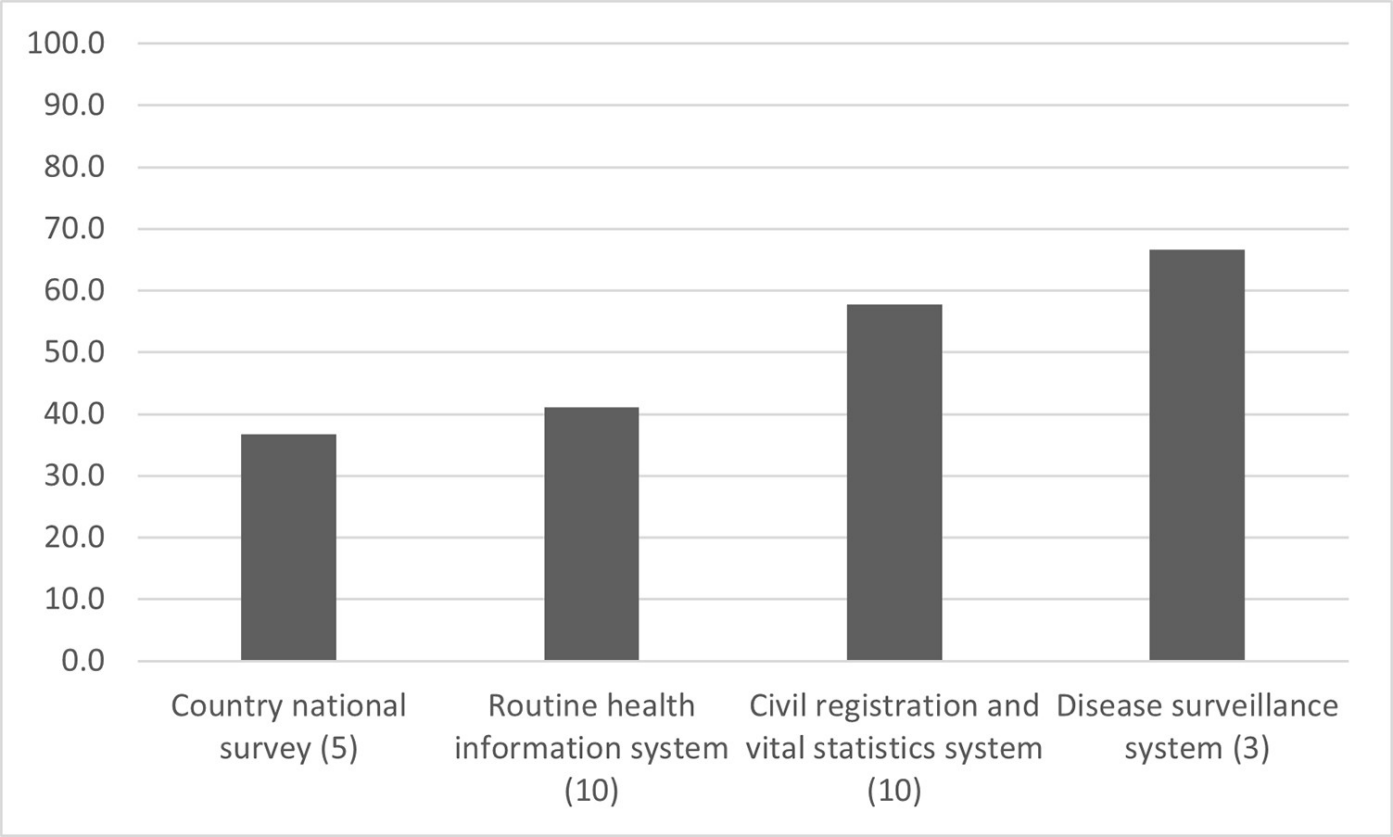
Percentage of SDG3 indicators reported in VNRs by data source. Note: Numbers in parenthesis are the number of SDG3 indicators.

Twenty-nine of the 32 SDG3 indicators can be derived from four key data sources of a well-functioning health information systems: civil registration and vital statistics (CRVS) system (11 indicators), country national survey (6), the disease surveillance system (3) and the routine health information system (9) (S1 Table). The remaining three, harmful alcohol use, official development assistance and IHR capacity are obtained from country administrative data, the Organization for Economic Cooperation and Development and key informant surveys and not necessarily from within the national health information system. The reporting of the health-related indicators by countries was much higher for the indicators derived from the disease surveillance system (66.7%) and CRVS system (67.8%) compared to those based on routine health information system (41.1%) and national surveys (36.7%, Fig 4).

## Discussion

This review of the voluntary national reviews from 18 countries of the EMR confirmed countries’ commitment to monitor progress on the SDGs with two of three reporting across all goals, particularly countries of higher income levels. The comprehensiveness of reporting progress on specific health-related indicators varied by type of indicator and by country income levels with maternal, neonatal and under-5 mortality rates the most commonly reported indicators. Limitations to monitoring progress on indicators were described in terms of national human resource capacity to collect and/or analyse information with a few mentioning issues like low data quality, lack of statistical tools, limited transparency in data dissemination, and lack of data disaggregation. Findings demonstrate capacity gaps: high proportion of data unavailable to measure progress, limited countries presented data for reporting progress and only one country reporting trends across all SDG 3 indicators. Given the importance of access to quality data to achieving the SDGs, significant work remains to ensure well-functioning national health information systems in countries in the region [8,9].

All reports demonstrated commitment to a multisectoral approach to collecting data by appointing a national authority for this task. Having a central authority is a key component of a strong governance structure but it is not enough. Achieving universal health coverage requires universal access to essential health information [18,19]. Legislation, regulations, policies, strategies and standards that establish clear roles and responsibilities to all involved promote data integration of different information system and regulate access to the information [20]. It also requires increasing investment in health information systems. Adopting an open access approach to sharing data can make health related data collected by various sectors more widely available and maximize their usage for policy-making [9,21]. Moving forward developing an investment framework defining the roles and responsibilities of relevant sectors to avoid duplication and updating legislations and policies is a key step to improving functionality of national health information systems [20,22,23].

Comprehensiveness of reporting was similar between middle-and high-income countries for mortality, morbidity, and means related indicators with the highest proportion being only 60%; middle income countries also had higher levels of reporting for risk factors and determinants indicators. Regionally, evidence indicates that barely two-thirds of births are registered, slight over half of death are registered, whereas six of 22 countries have sustainable capacity for public health surveillance and barely half have conducted at least one survey within the last 10 years (the lowest among all WHO regions) [24]. Only half of the countries in our assessment reported a country level analysis on data availability, with only four reporting for each SDG; such an analysis is useful to identify data gaps and strategies to address them. Data availability is a known challenge for monitoring regional progress on the health-related SDGs; for although two or more data points are available for a good portion of the indicators reported to WHO, the latest data for around 20% is four years or older [7]. This challenge is not unique but was also reflected in a review conducted in Europe along with concerns about limited analytical capacity, poor investment in technology and systems, and weak monitoring and evaluation [13]. Similarly was the lack of disaggregated data across all reports in this assessment as seen in the European review [13].

Collecting a small set of locally-relevant and policy-oriented indicators is key for successful implementation of health plans; this requires engaging across sectors to improve statistical capacity and increase data quality and availability [6,12,25]. Countries should focus their efforts in building national capacity across all data sources, particularly CRVS and population-based surveys.

CRVS systems are a foundation for good governance; birth and death registration are essential for estimating disease burden and for assessing the impact and cost-effectiveness of public health programmes and health interventions [26]. Ten of the 50 regional core indicators depend on a well-developed CRVS system. Although some progress has been made in completeness of vital event registration since 2015 when the region endorsed a strategy to strengthen their CRVS system [27,28], a large portion of births and deaths remain unregistered [29]. Various policy approaches that include both supply and demand components based on country’s health system governance and sociocultural context, can improve registration. Increasing public awareness on the importance of birth and death registration, introducing electronic death notification platforms and digital notification systems, and periodic evaluations of the quality of medical certification of causes of death, are some of the priority actions recommended by WHO to improve this system in countries of the region [20]. Documenting national policies that help strengthen systems in countries of the region would provide useful evidence in shaping regionally relevant policies and operational considerations for strengthen CRVS systems in the EMR [26].

National population-based surveys are critical components for a health information system supportive for SDGs; particularly so countries can have locally derived information rather than depend on international estimations [12]. They complement CRVS systems as a key source of information to monitor trends and assess inequality. A large majority of the regional core indicators could be generated from national population-based surveys including the health examination survey, Demographic Health Survey, Multiple Indicator Cluster Surveys and the Global School Health Survey [30]. These surveys, conducted over a 10 year period, could generate more robust data than available from routine systems currently available in the region [30]. Developing and implementing a national survey plan would enable a country to obtain a good portion of health-related SDG 3 indicators [20].

The country reports attributed limited data availability to challenges in data collection, quality, access, analysis and interpretation. Human and financial resources are common challenges associated with health information systems. Lack of data storage and standardization limits data collection, lack of standardization, training and equipment may inhibit data processing and analysis as well as data dissemination [31]. Monitoring the health-related SDGs requires a large variety of data systems including administrative systems, disease registries along with CVRS and household surveys [21]. Digital technology, a key transformation needed to meet the SDGs, provides an opportunity to strengthen collection and access to health information [10,18,32]. For example, DHIS2 (District Health Information System Version 2), a flexible open-source platform used in several countries of the region, leverages technology to collect, process, analyse and report aggregated information from the primary care facilities [10]. Routine health information systems along with CRVS provide a wealth of information about population health. A comprehensive assessment of the health information system can provide the basis for improving routine health information systems to better monitoring the health-related SDG targets and indicators [33].

Voluntary national reviews are highly formalized comprehensive reports; including an annex with data with gender statistics and disaggregated data, is encouraged but not required [2]. Being voluntary, providing details of specific targets and indicators for all 17 SDGs is not mandatory which would be a key reason why limited data is included in the reports. Additionally, as strategic documents, varying details on efforts made in data availability, quality, and coverage is understandable. Reporting at a high strategic level without providing comprehensive reporting on over 150 indicators does not necessarily indicate inability to report on specific indicators. At the same time, their preparation process results in a lengthy report with few if any references so interpretation is left to the subjective judgement of the authors.

## Conclusion

Voluntary national reviews from the countries in the EMR while demonstrating commitment to the SDGs recognized weak national health information systems, limited data availability and analytical capacity as challenges to monitoring and reporting. Strengthening health information systems regulatory frameworks, data collection capacities including strengthening CRVS systems and population-based surveys are key steps to enhancing access to quality data which in turn can contribute to achieving the health-related SDGs.

## Supporting information

S1 Table Reporting on SDG3 indicators in the Voluntary National Reviews (VNRs) in the Eastern Mediterranean Region, 2016–2021. (DOCX)

S2 Table Reporting of health-related SDG indicators in the VNRs. (DOCX)
