# Supplementary material for: Measuring progress on health and well-being in the Eastern Mediterranean Region via voluntary national reviews, 2016–2021: What does the data reveal?
Source: PLOS Glob Public Health. 2024 Jul 18;4(7):e0002838. doi: 10.1371/journal.pgph.0002838 (PMC11257290; doi:10.1371/journal.pgph.0002838)
Supplement: S1 Table — (DOCX) [file pgph.0002838.s001.docx]

**S1 Table. Reporting on SDG3 indicators in the Voluntary National Reviews (VNRs) in the Eastern Mediterranean Region, 2016-2021**

| **Country (year)*** | **3.1 Maternal mortality** | | **3.2 Child mortality** | | **3.3 Communicable Diseases** | | | | | | **3.4 NCDs** | | **3.5 Alcohol** | **3.6 RTIs** | **3.7 Reproductive health** | | **3.8 UHC** | | | | **3.9 Pollution related mortality** | | | | **3.a FCTC** | | **3.b Immunization coverage and medicines** | | | | | **3.c Health workforce** | | | | **3.d IHR (2005)** |
| --- | --- | --- | --- | --- | --- | --- | --- | --- | --- | --- | --- | --- | --- | --- | --- | --- | --- | --- | --- | --- | --- | --- | --- | --- | --- | --- | --- | --- | --- | --- | --- | --- | --- | --- | --- | --- |
|  | 3.1.1 Maternal mortality ratio (per 100 000 live births)^a^ | 3.1.2 Births attended by skilled health personnel (%) | 3.2.1 Under-5 mortality rate (per 1000 live births)^a^ | 3.2.2 Neonatal mortality rate (per 1000 live births)^a^ | 3.3.1 New HIV infections (per 1000 uninfected people) | 3.3.2 TB incidence (per 100 000 population) | 3.3.3 Malaria incidence (per 1000 population at risk) | 3.3.4 Hepatitis B prevalence among children under 5 years of age (per 100 000 population) | 3.3.5 Number of people requiring interventions for leishmaniasis | 3.4.1 Probability of dying from NCD (between ages 30 and 69) (%)^a^ | | 3.4.2 Suicide mortality rate (per 100 000 population)^a^ | 3.5.2 Harmful alcohol use (litres of pure alcohol per capita ≥ 15 years of age) | 3.6.1 Mortality rate from road traffic injuries (per 100 000 population)^a^ | 3.7.1 Women of reproductive age (15–49 years) who had their need for family planning satisfied with modern methods (%) | 3.7.2 Adolescent birth rate (per 1000 women aged 15–19 years) | 3.8.1 UHC service coverage index^a^ | 3.8.2 Large expenditure on heath as a share of total household expenditure (> 25%) | 3.8.2 Large household expenditure as a share of total health care expenditure (> 10%) | 3.9.1 Mortality rate attributed to household and ambient air pollution (per 100 000 population) | | 3.9.2 Mortality rate attributed to unsafe water, unsafe sanitation and lack of hygiene (per 100 000 population) | 3.9.3 Mortality rate attributed to unintentional poisoning (per 100 000 population) | 3.a.1 Prevalence of tobacco use among persons 15 years and older (%) | | 3.b.1 DTP3 coverage (%) | | 3.b.1 MCV2 coverage (%) | 3.b.1 PCV3 coverage (%) | 3.b.2 Official development assistance for medical research per capita (US$) | 3.b.3 Availability of essential medicines in public health facilities (%) | 3.c.1 Density of dentists (per 10 000 population) | 3.c.1 Density of nurses (per 10 000 population) | 3.c.1 Density of pharmacists (per 10 000 population) | 3.c.1 Density of physicians (per 10 000 population) | 3.d.1 International Health Regulations (2005) capacity and health emergency preparedness |
| Afghanistan, 2021 | * | * | * | * | * | * | * |  |  |  | |  |  |  |  |  |  |  |  |  | |  |  |  | | * | |  |  |  |  |  |  |  |  |  |
| Bahrain, 2018 |  |  |  |  |  |  |  |  |  |  | | * |  |  |  |  |  |  |  |  | |  |  |  | |  | |  |  |  |  |  |  |  |  |  |
| Egypt, 2020 | * |  | * | * |  |  |  |  |  |  | |  |  | * |  |  | * |  |  |  | |  |  |  | |  | |  |  |  |  |  |  |  |  |  |
| Iraq, 2021 | * | * | * | * |  |  |  |  |  |  | | * |  | * |  |  |  |  |  |  | |  |  |  | |  | |  |  |  |  |  |  |  |  |  |
| Jordan, 2017 |  |  | * |  |  |  |  |  |  |  | |  |  |  |  |  |  |  |  |  | |  |  |  | |  | |  |  |  |  |  |  |  |  |  |
| Kuwait, 2019 |  |  |  |  |  |  |  |  |  |  | |  |  | * |  | * | * |  |  |  | |  |  |  | |  | |  |  |  | * |  |  |  |  |  |
| Lebanon, 2018 | * |  | * |  | * |  |  |  |  |  | |  |  | * |  |  | * |  |  |  | |  |  |  | |  | |  |  |  |  |  |  |  |  |  |
| Libya, 2020 |  |  |  |  |  |  |  |  |  |  | |  |  |  |  |  |  |  |  |  | |  |  |  | |  | |  |  |  |  |  |  |  |  |  |
| Morocco, 2020 | * | * | * | * | * | * | * | * | * | * | | * | * | * | * | * | * | * | * | * | | * | * | * | | * | | * | * | * | * | * | * | * | * | * |
| Oman, 2019 | * |  | * |  | * | * | * | * |  |  | |  |  | * |  | * |  |  |  |  | |  |  |  | | * | |  |  |  |  |  |  |  |  | * |
| Pakistan, 2019 |  | * | * | * |  |  |  |  |  |  | |  |  |  | * |  |  |  |  |  | |  |  |  | | * | |  |  |  |  |  |  |  |  |  |
| Palestine, 2018 |  |  |  |  |  |  |  |  |  |  | |  |  |  | * |  |  |  |  |  | |  |  |  | |  | |  |  |  |  |  |  |  |  |  |
| Qatar, 2021 | * | * | * | * | * | * | * | * | * | * | | * | * | * | * | * | * |  |  | * | | * | * | * | | * | | * | * | * | * | * | * | * | * | * |
| Saudi Arabia, 2018 |  |  |  |  |  |  |  |  |  |  | |  |  |  |  |  |  |  |  |  | |  |  |  | |  | |  |  |  |  |  |  |  |  |  |
| Sudan, 2018 | * | * |  |  |  |  |  |  |  |  | |  |  |  | * | * |  |  |  |  | |  |  |  | |  | |  |  |  |  |  |  |  |  |  |
| Syria, 2020 |  |  |  |  |  |  |  |  |  |  | |  |  |  |  |  |  |  |  |  | |  |  |  | |  | |  |  |  |  |  |  |  |  |  |
| Tunisia, 2021 |  |  |  |  |  |  |  |  |  |  | |  |  |  |  |  |  |  |  |  | |  |  |  | |  | |  |  |  |  |  |  |  |  |  |
| UAE, 2016 |  |  |  |  |  |  |  |  |  |  | |  |  |  |  |  |  |  |  |  | |  |  | * | |  | |  |  |  |  |  |  |  |  |  |

Note: Black: In VNR, Grey: Not in VNR; In Core Indicators; White: Not in VNR; Not in WHO regional core health indicators; *Indicators with trend data
