# Supplementary material for: Measuring progress on health and well-being in the Eastern Mediterranean Region via voluntary national reviews, 2016–2021: What does the data reveal?
Source: PLOS Glob Public Health. 2024 Jul 18;4(7):e0002838. doi: 10.1371/journal.pgph.0002838 (PMC11257290; doi:10.1371/journal.pgph.0002838)
Supplement: S2 Table — (DOCX) [file pgph.0002838.s002.docx]

**S2 Table. Reporting of health-related SDG indicators in the VNRs**

| **Country (year)*** | **1. Poverty** | **2. Nutrition** | | | **4. Education** | | **5. Gender equality** | | **6. Water and sanitation** | | **8. Employment** | | | | **11. Air Pollution** | | **16. Violence** | | |  |
| --- | --- | --- | --- | --- | --- | --- | --- | --- | --- | --- | --- | --- | --- | --- | --- | --- | --- | --- | --- | --- |
|  | 1.1.1 Proportion of population living below the international poverty line (%) | 2.2.1 Stunting among children under 5 (%) | 2.2.1 Wasting among children under 5 (%)^a^ | 2.2.2 Overweight among children under 5 (%) | 4.1.1 Net primary school enrolment ratio (per 100 school-age children) | 4.6.1 Literacy rate (15–24 years) (%) | 5.2.1 Proportion of ever-partnered women and girls aged 15 years and older subjected to violence (%) | 5.6.1 Proportion of women aged 15–49 years who make their own informed decisions regarding sexual relations, contraceptive use and reproductive health care (%) | 6.1.1 Access to improved drinking-water (%)^a^ | 6.2.1 Access to improved sanitation facilities (%)^a^ | | 8.5.2 Unemployment rate, both sexes (%) | 8.5.2 Unemployment rate, females (%) | 8.5.2 Unemployment rate, males (%) | | 11.6.2 Annual mean levels of fine particulate matter in cities (mg/m^3^) | | 16.1.2 Conflict-related deaths (per 100 000 population) | 16.2.1 Proportion of children aged 1–17 years who experienced any physical punishment and/or psychological aggression by caregivers (%) | |
| Afghanistan, 2021 |  |  |  |  |  |  |  |  |  |  | |  |  |  | |  | |  |  | |
| Bahrain, 2018 |  |  |  |  |  |  |  |  |  |  | |  |  |  | |  | |  |  | |
| Egypt, 2021 |  |  |  |  |  |  |  |  |  |  | |  |  |  | |  | |  |  | |
| Iraq, 2021 |  |  |  |  |  |  |  |  |  |  | |  |  |  | |  | |  |  | |
| Jordan, 2017 |  |  |  |  |  |  |  |  |  |  | |  |  |  | |  | |  |  | |
| Kuwait, 2019 |  |  |  |  |  |  |  |  |  |  | |  |  |  | |  | |  |  | |
| Lebanon, 2018 |  |  |  |  |  |  |  |  |  |  | |  |  |  | |  | |  |  | |
| Libya, 2020 |  |  |  |  |  |  |  |  |  |  | |  |  |  | |  | |  |  | |
| Morocco, 2020 |  |  |  |  |  |  |  |  |  |  | |  |  |  | |  | |  |  | |
| Oman, 2019 |  |  |  |  |  |  |  |  |  |  | |  |  |  | |  | |  |  | |
| Pakistan, 2019 |  |  |  |  |  |  |  |  |  |  | |  |  |  | |  | |  |  | |
| Palestine, 2018 |  |  |  |  |  |  |  |  |  |  | |  |  |  | |  | |  |  | |
| Qatar, 2021 |  |  |  |  |  |  |  |  |  |  | |  |  |  | |  | |  |  | |
| Saudi Arabia, 2018 |  |  |  |  |  |  |  |  |  |  | |  |  |  | |  | |  |  | |
| Sudan, 2018 |  |  |  |  |  |  |  |  |  |  | |  |  |  | |  | |  |  | |
| Syria, 2020 |  |  |  |  |  |  |  |  |  |  | |  |  |  | |  | |  |  | |
| Tunisia, 2021 |  |  |  |  |  |  |  |  |  |  | |  |  |  | |  | |  |  | |
| UAE, 2016 |  |  |  |  |  |  |  |  |  |  | |  |  |  | |  | |  |  | |

Note: Black: In VNR, Grey: Not in VNR; In Core Indicators; White: Not in VNR; Not in WHO regional core health indicators
